# Supplementary material for: The Influence of Hydrodynamic Conditions in a Laboratory-Scale Bioreactor on Pseudomonas aeruginosa Metabolite Production
Source: Microorganisms. 2022 Dec 29;11(1):88. doi: 10.3390/microorganisms11010088 (PMC9866481; doi:10.3390/microorganisms11010088)
Supplement: Supplementary file 1 [file microorganisms-11-00088-s001.zip › microorganisms-2095449-supplementary.pdf]

microorganisms

MDPI

Supplementary Materials

The Influence of Hydrodynamic Conditions in a Laboratory-Scale Bioreactor on *Pseudomonas aeruginosa* Metabolite Production

Maciej Konopacki [1,2,*], Joanna Jabłońska [1], Kamila Dubrowska [1], Adrian Augustyniak [1,3,4,*], Bartłomiej Grygorcewicz [2], Marta Gliźniewicz [2], Emil Wróblewski [1], Marian Kordas [1], Barbara Dołęgowska [2] and Rafał Rakoczy [1]

[1] Department of Chemical and Process Engineering, Faculty of Chemical Technology and Engineering, West Pomeranian University of Technology in Szczecin, Piastów Avenue 42, 71-065 Szczecin, Poland
[2] Department of Laboratory Medicine, Chair of Microbiology, Immunology and Laboratory Medicine, Pomeranian Medical University in Szczecin, Powstańców Wielkopolskich 72, 70-111 Szczecin, Poland
[3] Chair of Building Materials and Construction Chemistry, Technische Universität Berlin, Gustav-Meyer-Allee 25, 13355 Berlin, Germany
[4] Institute of Biology, University of Szczecin, Wąska 13 Str., 71-415 Szczecin, Poland
* Correspondence: maciej.konopacki@zut.edu.pl (M.K.); adrian.augustyniak@zut.edu.pl (A.A.)

S.1. Materials and methods

Measurements of viability, biomass and metabolites production

The viability of the cells was quantified employing resazurin assay that allows the monitoring of aerobic respiration of the cells. Briefly, 10 µL of 1 mg/mL resazurin PBS solution was added to each well containing 100 µL of the culture. The fluorescence was recorded at the wavelengths of 520 nm for excitation and 590 nm for emission.

The dry biomass was obtained by centrifuging the culture, drying the pellets and weighing biomass on the analytical balance.

The optical density, dry biomass, plate count, and viability assays were carried out simultaneously for 12 hours in a control culture cultivated at 37°C in 2 L of King A medium without agitation and aeration (see Fig. S1). It allowed showing the linear relationship between the optical density of the culture and dry biomass per millilitre which can be expressed as (adjusted $R^2 = 0.95802$) (see Fig. S2)

$$m_{dry} = 2.3429\, OD - 0.0521 \tag{S1}$$

where: m_{dry} – dry biomass, mg·mL^{-1}; OD – optical density.

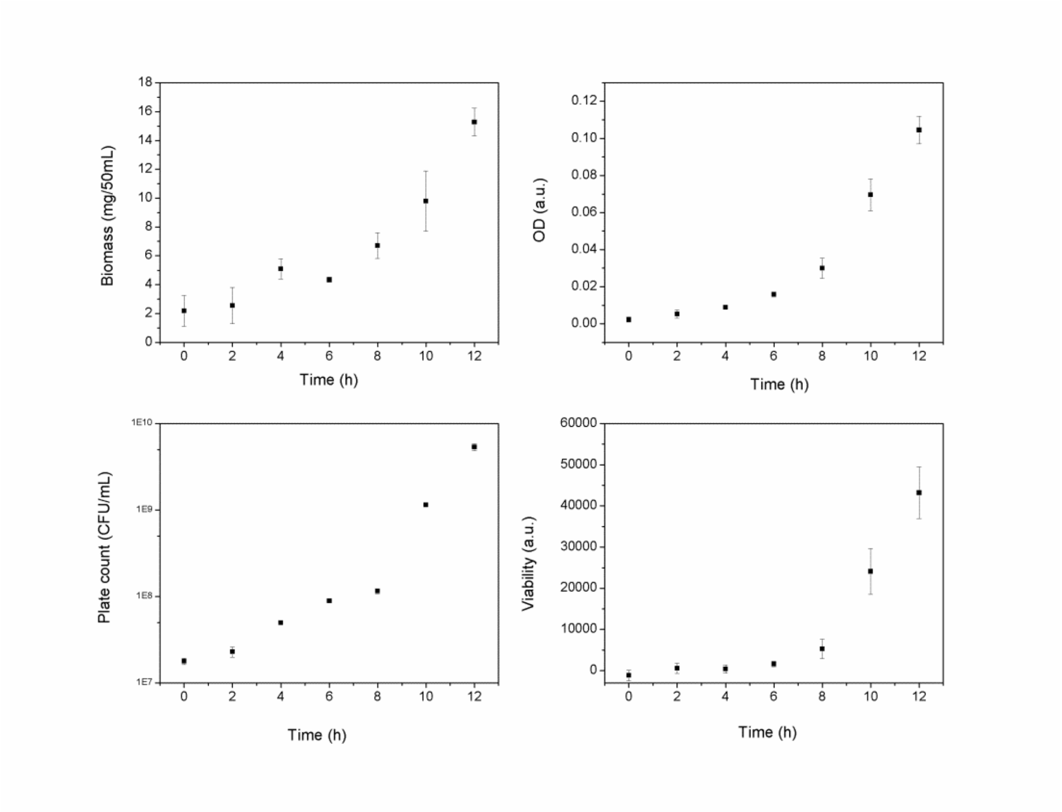

Figure S1. The monitoring of dry biomass, OD, plate count and viability.

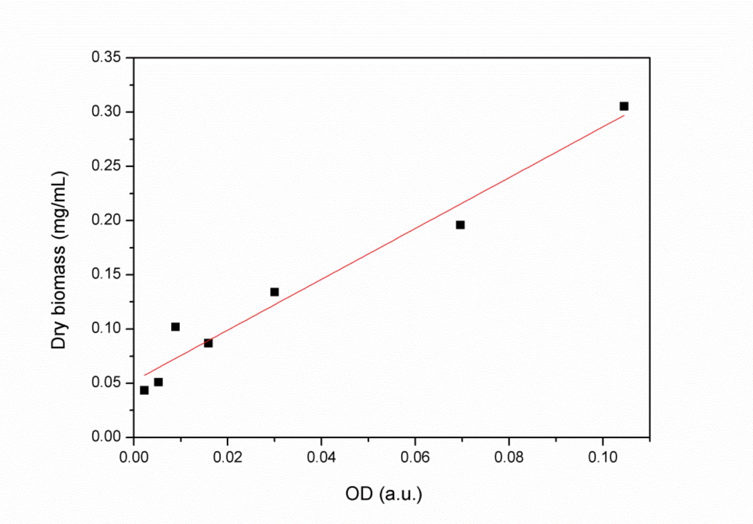

Figure S2. The linear relation between dry biomass and OD.

To accurately calculate the concentration of pyocyanin and rhamnolipids calibration curves of these substances were prepared using HPLC-grade reagents (Fig. S3 and Fig. S4, respectively).

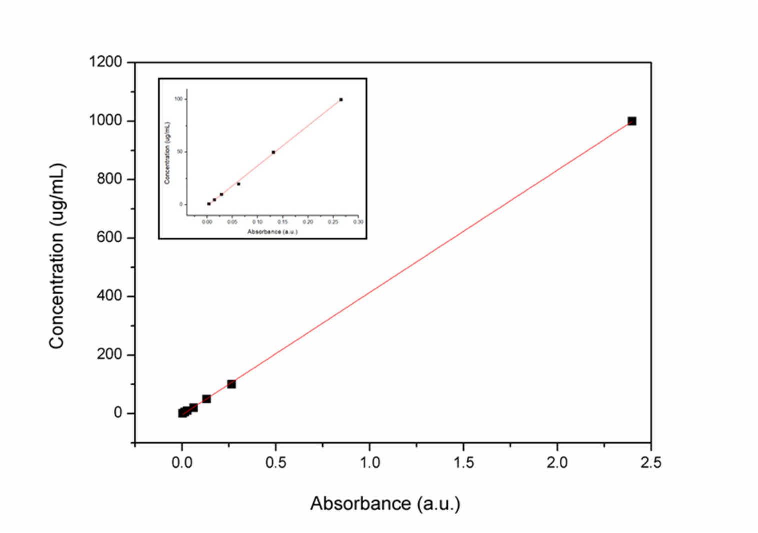

Figure S3. Calibration curve of pyocyanin: PYO = 418.31 × absorbance - 3.93 (adjusted R^2 = 0.99988; inlet shows the curve for lower concentrations).

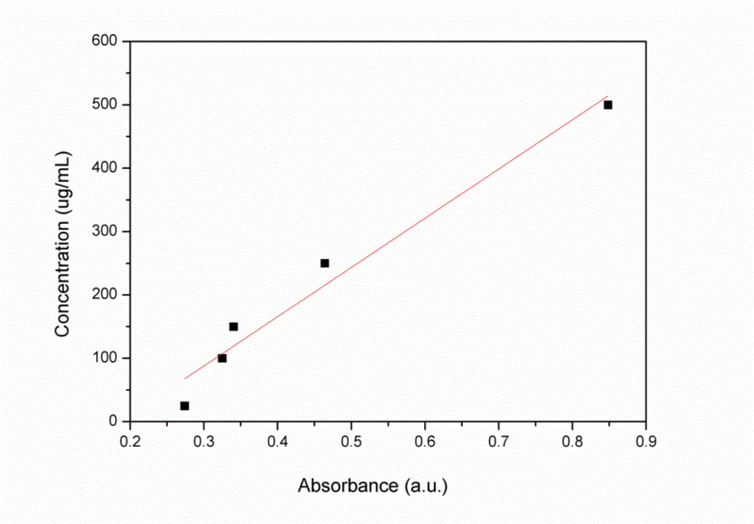

Figure S4. Calibration curve of rhamnolipids: Rhamnolipids = 776.92275 × absorbance - 144.87421 (adjusted R^2 = 0.95848).

S.2. Power consumption for ungassed and gassed conditions

Bioreactor system performance can be described using a power consumption per medium volume. This parameter is often used for the system scale-up and is proportional to the impeller speed:

$$\left(\frac{P}{V}\right) \propto n \quad \Rightarrow \quad \left(\frac{P}{V}\right) = p_1\, n^{p_2} \qquad \text{(S2)}$$

where: P - power consumption, W; V – volume, m^3; n - impeller speed, s^{-1}

Moreover, the system power characteristics are used to compare the effectiveness of various systems. It is defined as a power number *Ne* as a function of Reynolds number *Re*:

$$\mathrm{Ne} = f(\mathrm{Re}) \quad \Rightarrow \quad \left(\frac{P}{\rho\, n^3\, d^5}\right) = f\left(\frac{n\, d^2}{\nu}\right) \qquad \text{(S3)}$$

where: d - impeller diameter, m; ν - kinematic viscosity, $m^2\ s^{-1}$

Results of specific power consumption and power characteristics are presented in Fig. S.5.

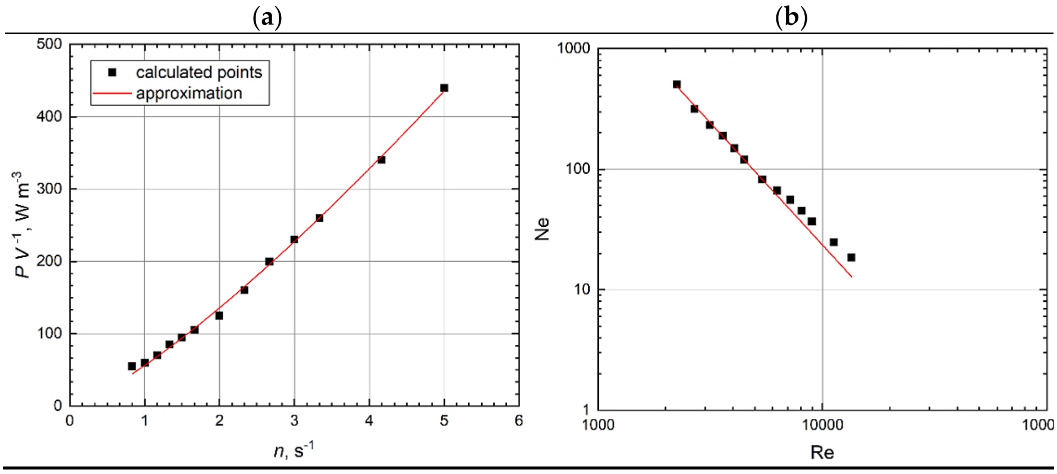

Figure S5. Power consumption for ungassed conditions: a) specific power consumption, b) power characteristics.

For tested conditions in a bioreactor, the specific power consumption has the form of $P/V = 60.07n^{1.2035}$. Specific power consumption is increasing with the impeller speed. Moreover, the power number decreased with the Reynolds number, which is expected for Rushton-type turbine within the transient range of flow.

For the bioreactor with aeration, the real energy input is defined as the ratio between the power consumption for gassed and ungassed conditions. In general, this ratio is proportional to the inverted flow number *Q*, which is defined as follows:

$$\left(\frac{P_g}{P}\right) \propto \mathrm{Q}^{-1} \quad \Rightarrow \quad \left(\frac{P_g}{P}\right) \propto \left(\frac{\dot{V}_g}{n d^3}\right)^{-1} \qquad \text{(S4)}$$

where P_g - power consumption for gassed conditions W m^{-3}, $\dot{V}_g$ - volumetric flow rate of gas m^3 s^{-1}

The calculated ratio P_g / P as a function of flow number *Q* is illustrated in Fig. S.6.

Obtained data shows that the power consumption for gassed conditions is lower than for ungassed conditions. For low gas flow rate (small flow number) those differences are rather slight but with increasing flow rate of gas relative power consumption drops faster, showing significant changes.

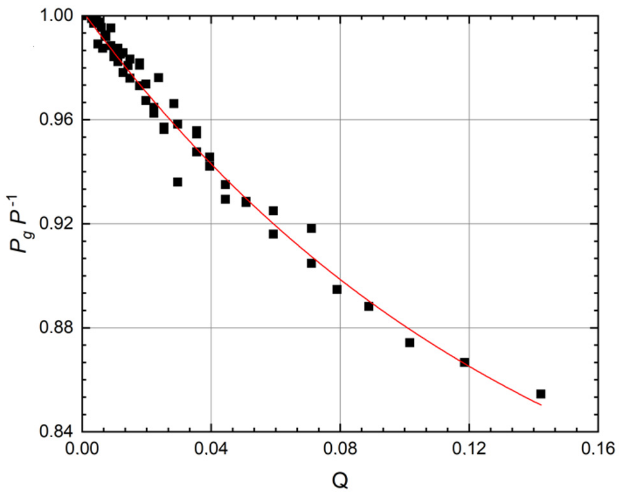

Figure S6. Relative power consumption as a function of flow number for the tested bioreactor.

S.3. Mixing time for ungassed and gassed conditions

The result of mixing time τ_{95} measurements, both for ungassed and gassed conditions can be presented as a correlation to the Reynolds number:

$$n\tau_{95} \propto \mathrm{Re} \tag{S5}$$

Experimental results of mixing time in the bioreactor are presented in Fig. S.7.

(**a**) (**b**)

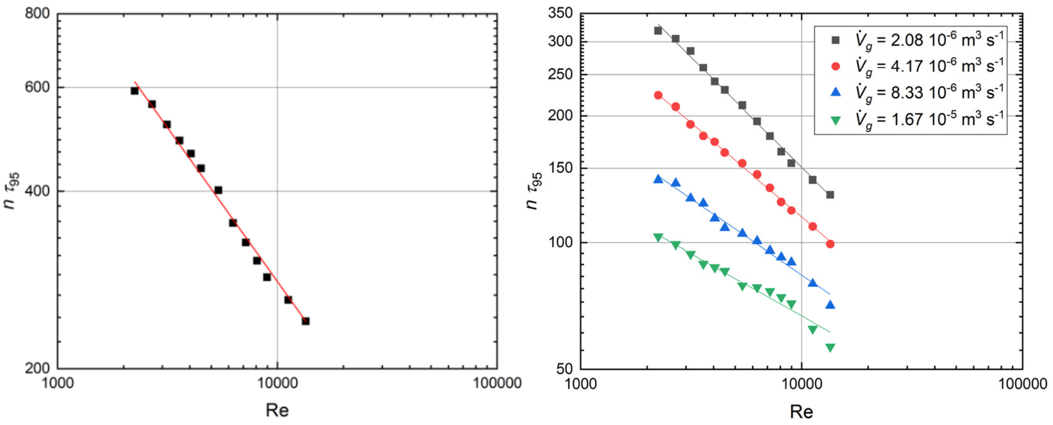

Figure S7. Measured mixing time in a bioreactor: a) ungassed, b) gassed conditions.

For all studied cases mixing time is decreasing with the raising impeller speed. Mixing time is also strongly affected by gas flow rate (GFR). Additional movement of liquid created by gas bubbles increases the mixing of liquid particles decreasing measured mixing time with increasing GFR. The aeration process improves mixing in the bioreactor up to six times for the highest GFR, especially for a very low range of impeller speed. This situation can be very useful to perform a mixing process without creating high-shear stress regions by the high-speed impeller blade.

S.3. Mass transport (aeration)

Mass transport evaluation was based on the concentration of dissolved oxygen in the bioreactor medium. Changes in oxygen concentration in the time allowed to calculate the volumetric gas-liquid mass transfer coefficient $k_L a$ using the following formula:

$$k_L a = \frac{1}{t}\ln\left(\frac{c^* - c_{fin}}{c^* - c_{in}}\right) \quad \text{(S6)}$$

where: t - process time, s; c^* - equilibrium concentration, kg m^{-3}; c_{fin} - final concentration, kg m^{-3}; c_{in} - initial concentration, kg m^{-3}

The calculated $k_L a$ coefficient was presented for various mixing speed and gas flow rate in the Table S1 and as a function of specific power consumption for gassed conditions and illustrated in Fig. S.8 for various gas flow rates.

Table S1. $k_L a$ [s^{-1}] values for various mixing speed and gas flow rate.

N [RPM]	*Vg = 1000 ccm*	*Vg = 500 ccm*	*Vg = 250 ccm*	*Vg = 125 ccm*
50	0.0021	0.0015	0.0006	0.00023
60	0.0025	0.0016	0.0006	0.00024
70	0.0027	0.0016	0.0007	0.00028
80	0.0028	0.0018	0.0007	0.00033
90	0.0029	0.002	0.0009	0.00036
100	0.0029	0.0021	0.001	0.00039
120	0.003	0.0022	0.0011	0.00044
140	0.0034	0.0023	0.0012	0.00051
160	0.0041	0.0025	0.0013	0.00062
180	0.0044	0.0028	0.0015	0.00068
200	0.005	0.0031	0.0017	0.00075
250	0.006	0.0039	0.0021	0.001
300	0.0068	0.0046	0.0026	0.0012

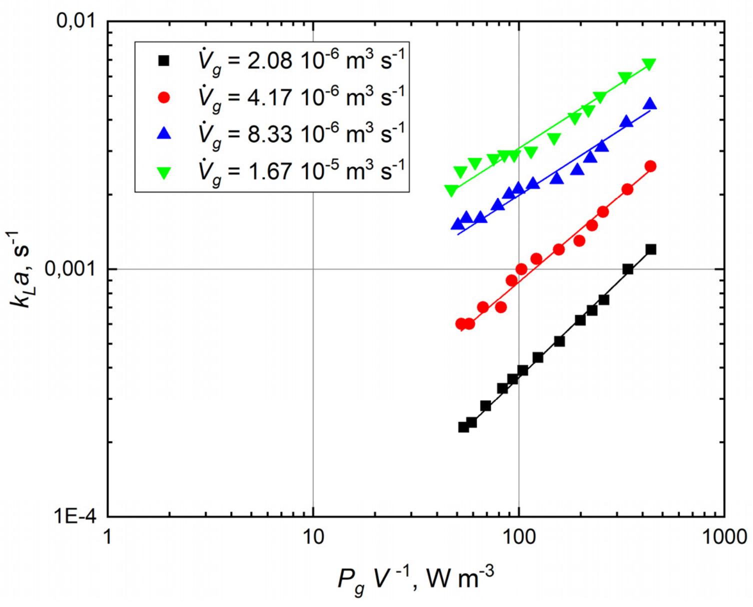

Figure S8. Changes of $k_L a$ coefficient in the function of specific power consumption.

The presented data confirm that the $k_L a$ coefficient depends strongly on the GFR, resulting in maximal values for the highest GFR. Moreover, it is raising with the increasing specifying power consumption. This could be connected to improved gas bubbles dispersion in the bioreactor medium and breakage of bigger bubbles into a few small ones, which increase mass transfer interface area, improving the total process.

S.5. CFD analysis

The Computational Fluid Dynamics (CFD) analysis was performed to study hydrodynamics inside the working bioreactor. In the beginning, a 3D model of fluid geometry was created using AutoCAD 2022 software and then implemented in ANSYS Design Modeler. Then a numerical grid was created using the ANSYS Meshing. A typical grid was composed of an approx. 300 thousand tetrahedral elements with 0.7 mean orthogonal quality. Such a numerical grid was introduced to the ANSYS CFX solver, where the boundary conditions and physics were specified. The multiple zones modelling with the frozen rotor model (FRM) was employed. This approach requires breaking up the model into multiple zones - stationary and rotating around the impeller and then an appropriate interface between these zones. The results in FRM are obtained for the steady state of bioreactor work. Moreover, the k-omega turbulence model was employed to represent the physical behaviour of fluid in motion. A typical result of the CFD analysis is presented in form of fluid velocity contours in bioreactor horizontal and vertical cross-sections for selected impeller speed, illustrated in Fig. S.9 - S.12, respectively.

(**a**) (**b**)

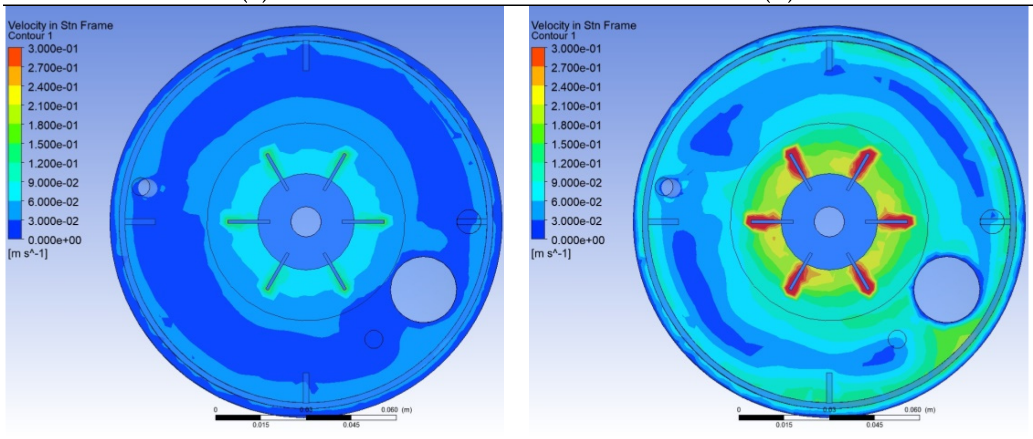

(**c**) (**d**)

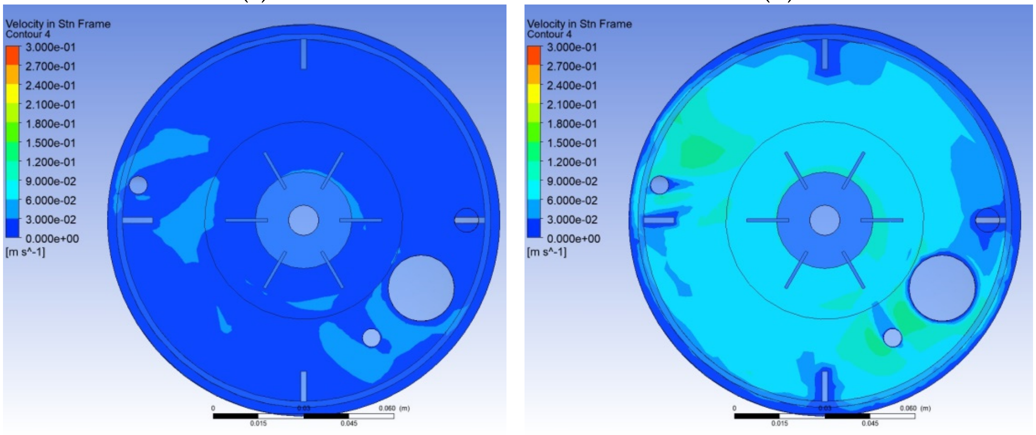

(**e**) (**f**)

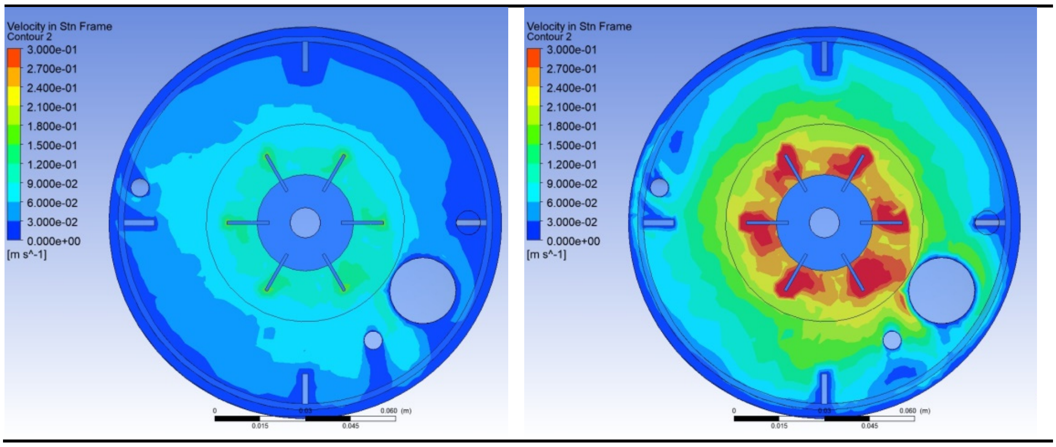

Figure S9. Velocity contours at horizontal cross-sections: a) at the lower impeller 60 rpm (Re_{mix} = 2695.89, b) at the lower impeller 160 rpm (Re_{mix} = 7189.04), c) between the impellers 60 rpm, d) between the impellers 160 rpm, e) at the upper impeller 60 rpm, f) at the upper impeller 160 rpm.

(**a**) (**b**)

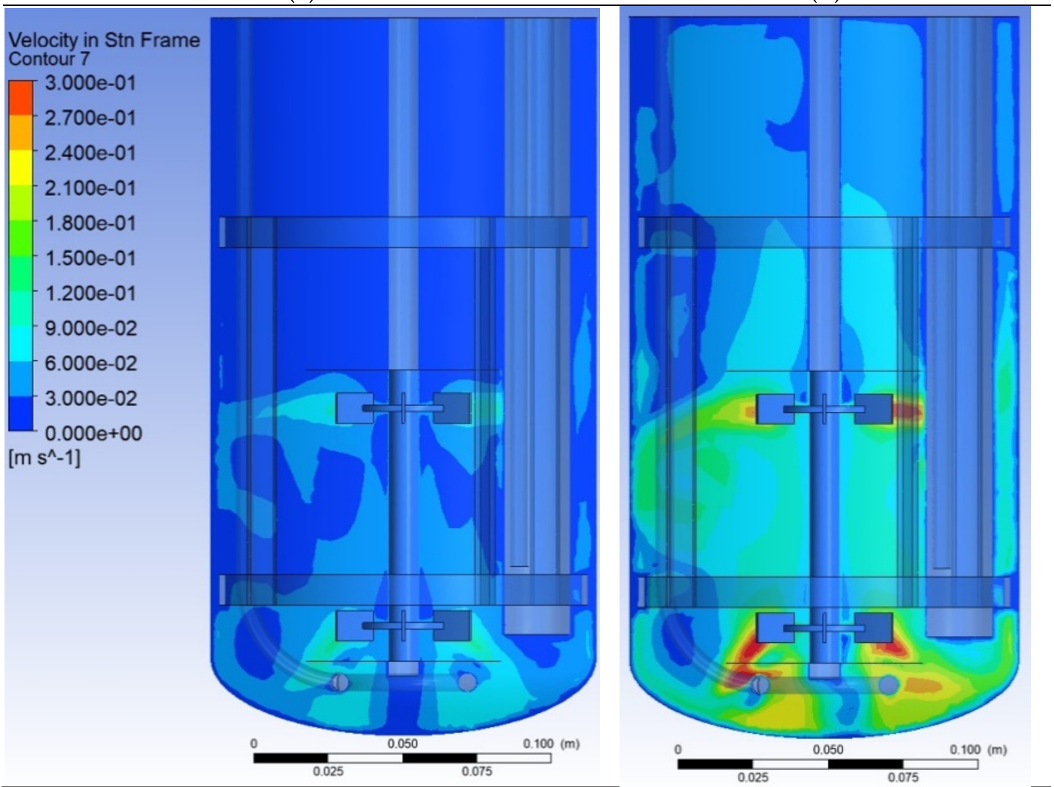

Figure S10. Velocity contours at the vertical cross-section: a) 60 rpm, b) 160 rpm.

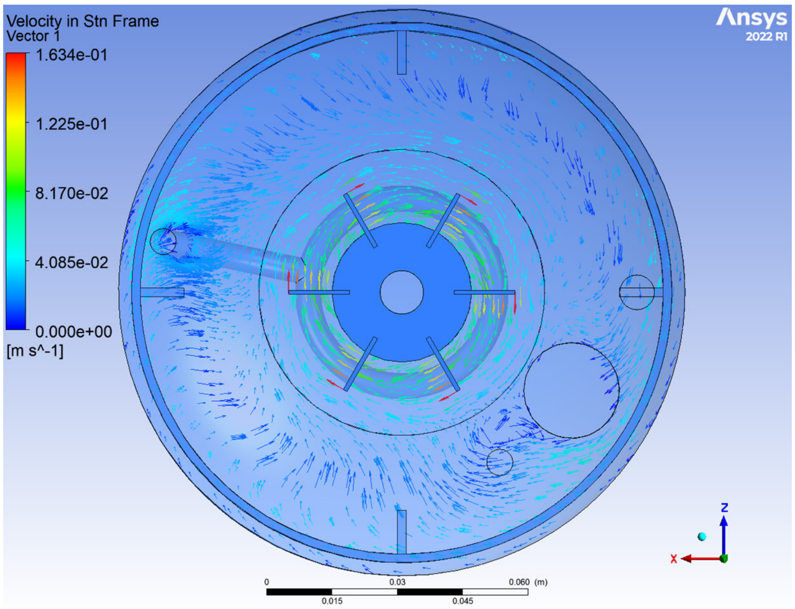

Figure S11. Fluid flow pattern around the turbine at 60 rpm

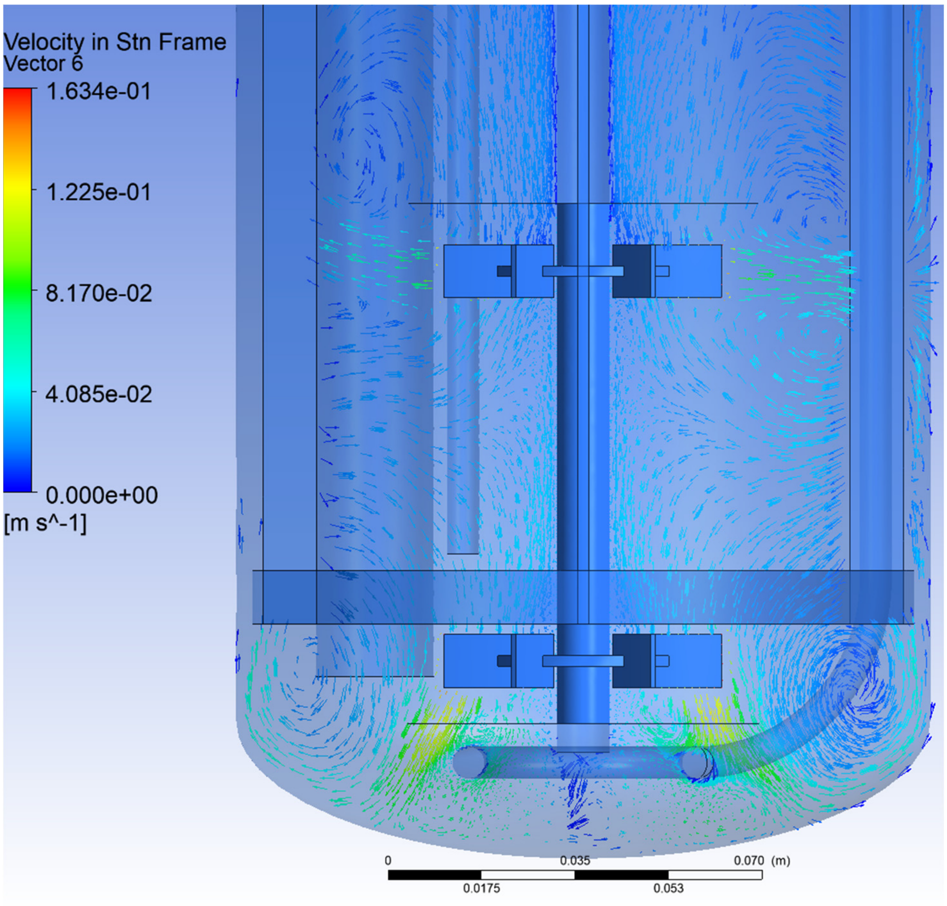

Figure S12. Fluid flow pattern in the axial cross-section

S.6. Growth factor estimation

Obtained growth curves were described by the logistic function given by the formula:

$$y(t) = \frac{a}{1 + \exp(b - c\ t)} \quad \text{(S7)}$$

Based on adjusted *a, b,* and *c* parameters it is possible to estimate the following parameters:

Maximum specific growth rate

$$\mu_{\max} = \frac{ac}{4} \quad \text{(S8)}$$

Lag-phase duration

$$\lambda = \frac{b-2}{c} \quad \text{(S9)}$$

Moreover, an asymptote of logistic function given by parameter *a* indicates the maximal concentration of biomass that can be achieved during the cultivation. Obtained data for all performed experiments are listed in the table below:

Table S2. Growth parameters estimated for various hydrodynamic conditions.

Re_{mix}	Re_g	a	μ_{max}	λ	φ	Φ
7189.04	41.02	0.2832	0.03427	3.894	0.1006	0.0513
2695.89	41.02	0.4400	0.09154	3.865	0.2957	0.2343
0	41.02	0.3043	0.04438	2.744	0.2098	0.1150
2695.89	20.51	0.5553	0.08827	4.735	0.2120	0.2120
0	20.51	0.4446	0.04777	4.507	0.0673	0.0538
7189.04	20.51	0.2826	0.02730	3.274	0.0788	0.0401
0	0	0.1221	0.02700	4.381	0.0738	0.0162
2695.89	0	0.1380	0.01894	5.454	0.0224	0.0056
7189.04	0	0.1167	0.02063	4.276	0.0608	0.0128

where: Re_{mix} – Reynolds number for mixing [-], Re_g – Reynolds number for gas flow [-], a – maximal concentration of biomass in term of OD [-], μ_{max} – maximal specific growth rate [hr^{-1}], λ – lag-phase duration time [hr], φ growth kinetics factor [-], Φ – growth factor [-].
